# Probing the Statistical Properties of Unknown Texts: Application to the Voynich Manuscript

**DOI:** 10.1371/journal.pone.0067310

**Published:** 2013-07-02

**Authors:** Diego R. Amancio, Eduardo G. Altmann, Diego Rybski, Osvaldo N. Oliveira, Luciano da F. Costa

**Affiliations:** 1 Institute of Physics of São Carlos, University of São Paulo, São Carlos, São Paulo, Brazil; 2 Max Planck Institute for the Physics of Complex Systems (MPIPKS), Dresden, Germany; 3 Potsdam Institute for Climate Impact Research (PIK), Potsdam, Germany; University of Maribor, Slovenia

## Abstract

While the use of statistical physics methods to analyze large corpora has been useful to unveil many patterns in texts, no comprehensive investigation has been performed on the interdependence between syntactic and semantic factors. In this study we propose a framework for determining whether a text (e.g., written in an unknown alphabet) is compatible with a natural language and to which language it could belong. The approach is based on three types of statistical measurements, i.e. obtained from first-order statistics of word properties in a text, from the topology of complex networks representing texts, and from intermittency concepts where text is treated as a time series. Comparative experiments were performed with the New Testament in 15 different languages and with distinct books in English and Portuguese in order to quantify the dependency of the different measurements on the language and on the story being told in the book. The metrics found to be informative in distinguishing real texts from their shuffled versions include assortativity, degree and selectivity of words. As an illustration, we analyze an undeciphered medieval manuscript known as the Voynich Manuscript. We show that it is mostly compatible with natural languages and incompatible with random texts. We also obtain candidates for keywords of the Voynich Manuscript which could be helpful in the effort of deciphering it. Because we were able to identify statistical measurements that are more dependent on the syntax than on the semantics, the framework may also serve for text analysis in language-dependent applications.

## Introduction

Methods from statistics, statistical physics, and artificial intelligence have increasingly been used to analyze large volumes of text for a variety of applications [1]–[11] some of which are related to fundamental linguistic and cultural phenomena. Examples of studies on human behaviour are the analysis of mood change in social networks [1] and the identification of literary movements [3]. Other applications of statistical natural language processing techniques include the development of statistical techniques to improve the performance of information retrieval systems [12], search engines [13], machine translators [14], [15] and automatic summarizers [16]. Evidence of the success of statistical techniques for natural language processing is the superiority of current corpus-based machine translation systems in comparison to their counterparts based on the symbolic approach [17].

The methods for text analysis we consider can be classified into three broad classes: (i) those based on first-order statistics (such as arithmetic mean and standard deviation) where data on classes of words are used in the analysis, e.g. frequency of words [18]; (ii) those based on metrics from networks representing text [3], [4], [8], [9], [19], where adjacent words (represented as nodes) are directionally connected according to the natural reading order; (iii) those using intermittency concepts and time-series analysis for texts [4]–[7], [20]–[23]. One of the major advantages inherent in these methods is that no knowledge about the meaning of the words or the syntax of the languages is required. Furthermore, large corpora can be processed at once, thus allowing one to unveil hidden text properties that would not be probed in a manual analysis given the limited processing capacity of humans. The obvious disadvantages are related to the superficial nature of the analysis, for even simple linguistic phenomena such as lexical disambiguation of homonymous words are very hard to treat. Another limitation in these statistical methods is the need to identify the representative features for the phenomena under investigation, since many parameters can be extracted from the analysis but there is no rule to determine which are really informative for the task at hand. Most significantly, in a statistical analysis one may not even be sure if the sequence of words in the dataset represents a meaningful text at all. For testing whether an unknown text is compatible with natural language, one may calculate measurements for this text and several others of a known language, and then verify if the results are statistically compatible. However, there may be variability among texts of the same language, especially owing to semantic issues.

In this study we combine measurements from the three classes above and propose a framework to determine the importance of these measurements in investigations of unknown texts, regardless of the alphabet in which the text is encoded. The statistical properties of words and the books were obtained for comparative studies involving the same book (New Testament) in 15 languages and distinct pieces of text written in English and Portuguese. The purpose in this type of comparison was to identify the features capable of distinguishing a meaningful text from its shuffled version (where the position of the words is randomized), and then determine the proximity of pieces of text.

As an application of the framework, we analyzed the famous Voynich Manuscript (VMS), which has remained indecipherable in spite of attempts from renowned cryptographers for a century. This manuscript dates back to the 15th century, possibly produced in Italy, and was named after Wilfrid Voynich who bought it in 1912. In the analysis we make no attempt to decipher VMS, but we have been able to verify that it is compatible with natural languages, and even identified important keywords, which may provide a useful starting point toward deciphering it.

## Results and Discussion

Here we report the statistical analysis of different measurements 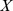 across different texts and languages. Each 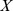 characterizing the whole text (book), being obtained from statistical analysis on the level of words, and normalized to the value obtained by the corresponding shuffled text (i.e., only values 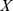 significantly different from 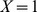 provide useful information). In some cases, 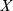 was obtained as an average over the values 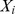 of different words 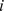 (e.g., the clustering coefficient 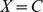). For these measurements, besides the average over all words 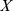 we considered also the average 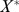 over the 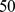 most frequent words. The detailed description of the different measurements 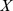 is found in the “Materials and Methods” Section, for the list of the 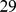 used measurements see the first column of Table 1.

**Table 1:** Statistical properties of measurements extracted from texts.

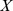	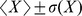			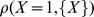			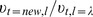		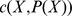		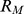	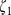	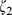	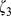	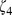
	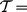 new	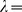 en	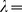 pt	new	en	pt	en	pt	en	pt					
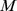 Vocabulary	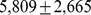	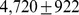	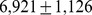	–	–	–	3.12	2.82	0.00	0.00	+1.00	–	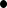	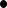	
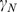 Zipf exponent	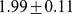	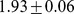	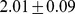	–	–	–	1.71	1.25	0.00	0.00	+0.86	–	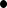		
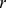 Assortativity	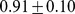	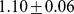	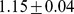	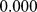	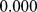	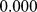	2.18	3.41	0.07	0.14	+0.07	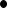	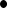	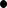	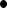
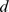 Diameter	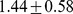	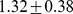	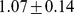	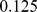	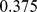	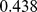	1.41	3.16	0.00	0.00	+0.08		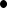		
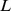 Shortest paths	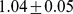	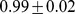	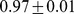	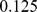	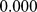	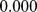	2.07	7.57	0.76	0.68	+0.20		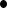	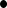	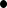
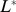 Shortest paths	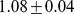	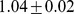	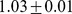	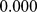	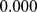	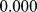	2.23	2.91	0.80	0.51	+0.34	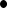	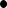	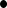	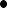
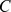 Clustering	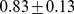	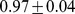	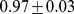	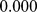	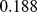	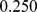	3.31	4.74	0.65	0.62	−0.34		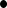	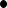	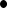
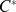 Clustering	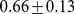	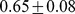	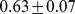	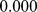	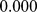	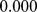	1.52	1.71	0.91	0.80	−0.58	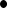	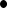		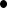
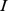 Intermittency	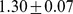	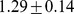	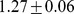	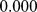	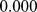	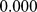	0.47	1.03	0.59	0.45	−0.43	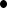			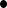
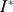 Intermittency	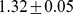	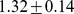	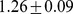	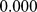	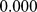	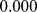	0.36	0.75	0.77	0.95	−0.26	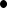		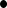	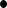
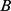 Betweenness	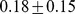	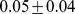	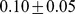	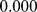	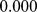	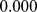	1.01	11.4	0.95	0.32	+0.27	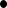	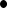		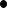
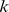 Degree	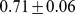	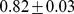	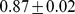	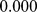	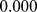	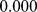	1.44	3.99	0.00	0.01	+0.53	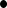	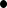	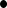	
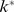 Degree	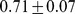	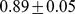	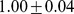	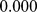	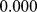	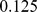	1.93	2.81	0.01	0.01	+0.26		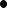	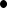	
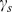 Selectivity exp.	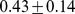	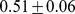	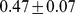	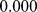	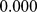	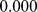	2.53	2.26	0.88	0.69	−0.49	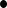	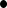	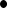	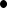
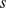 Selectivity	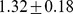	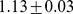	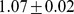	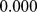	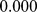	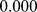	5.06	8.30	0.05	0.25	−0.51	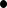	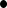	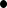	
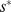 Selectivity	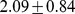	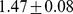	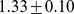	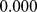	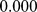	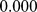	7.18	5.60	0.48	0.62	−0.39	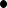	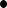	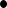	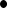
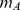 Network motif	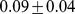	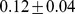	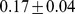	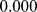	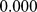	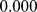	1.31	1.85	0.00	0.00	+0.02	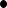	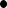		
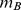 Network motif	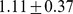	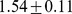	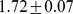	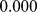	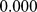	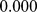	3.75	7.67	0.00	0.00	−0.09	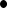	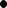	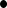	
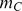 Network motif	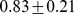	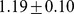	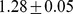	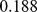	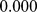	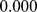	2.30	6.04	0.00	0.00	+0.04		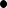	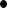	
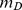 Network motif	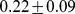	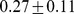	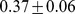	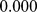	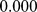	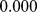	0.97	2.45	0.00	0.00	+0.24	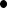	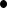		
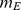 Network motif	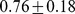	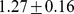	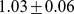	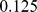	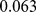	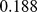	1.66	0.72	0.00	0.00	−0.23		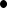		
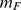 Network motif	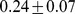	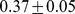	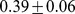	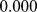	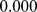	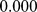	1.87	1.80	0.00	0.00	−0.20	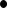	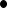		
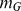 Network motif	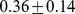	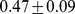	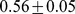	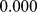	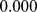	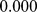	1.82	4.43	0.00	0.00	+0.14	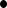			
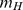 Network motif	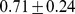	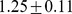	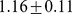	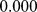	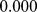	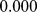	2.67	3.66	0.00	0.00	−0.17	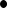	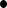	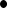	
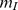 Network motif	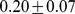	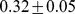	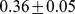	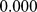	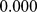	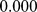	1.68	2.48	0.00	0.00	−0.14	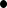	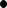		
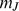 Network motif	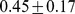	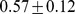	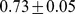	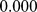	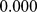	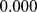	1.76	5.19	0.00	0.00	+0.11	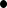	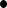		
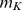 Network motif	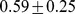	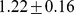	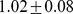	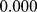	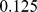	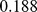	2.55	5.29	0.00	0.00	−0.24		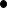	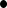	
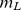 Network motif	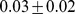	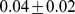	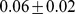	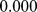	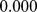	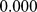	1.53	1.85	0.04	0.35	+0.10	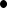	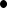		
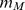 Network motif	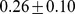	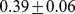	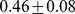	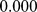	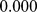	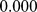	2.11	2.16	0.00	0.00	−0.14	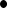	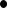	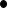	

Verification of which measurements satisfy conditions 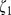, 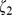, 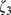 and 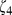. 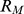 is the Pearson correlation between 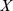 and the vocabulary size 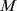. The measurements 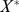 were obtained as an average over the 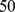 most frequent words, in contrast to the corresponding 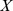 measurements which were obtained as an average over all words. We assume that 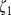, 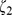, 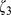 and 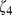 are satisfied respectively when 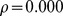, 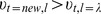, 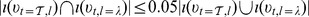 and 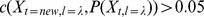. Measurements satisfying conditions for all three sets of texts are marked with a filled circle (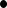).

### Variability across Languages and Texts

**Figure 1:**
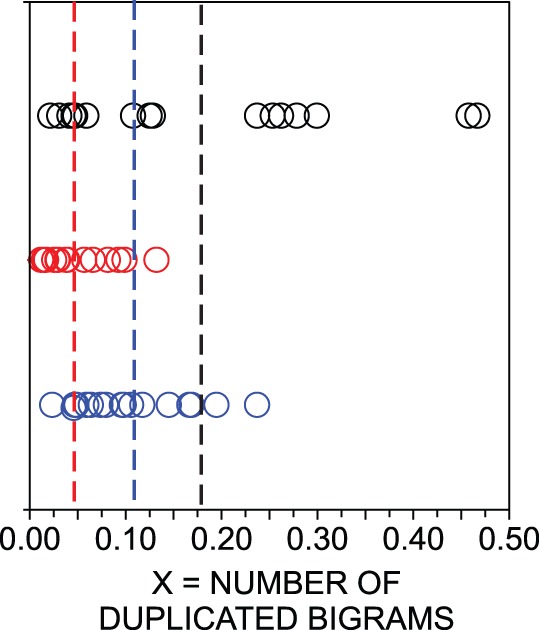
Distribution of the number of times words appear two times in a row (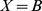) compared with the expected value in shuffled texts. Each circle represents a book (black, for distinct languages of the New Testament; red, for novels in English; and blue, for novels in Portuguese). The average 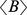 for the three sets of texts is represented as dashed lines. Note that all normalized values are far from 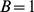, which suggests that 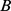 computed in natural languages is useful to distinguish shuffled, meaningless texts from documents written in a natural language.

The measurements described in this paper vary from language to language due to the syntactic properties. In a given language, there is also an obvious variation among texts on account of stylistic and semantic factors. Thus, in a first approximation one may assume that variations across texts of a measurement 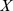 occur in two dimensions. Let 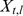 denote the value of 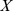 for text 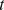 written in language 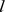. If we had access to the complete matrix 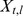, i.e. if all possible texts in every possible language could be analyzed, we could simply compare a new text 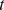 to the full variation of the measurements 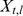 in order, e.g., to attribute to which languages 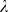 the text is compatible with. In practice, we can at best have some rows and columns filled and therefore additional statistical tests are needed in order to characterize the variation of specific measurements. For different texts, 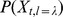 denotes the distribution of measurement 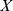 across different texts in a fixed language 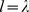 and 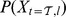 the distribution of 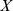 across a fixed text 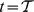 written in various languages. Accordingly, 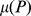 and 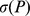 represent the expectation and the variation of the distribution 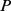. For concreteness, Figure 1 illustrates the distribution of 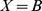 (number of times words appear two times in a row) for the three sets of texts we use in our analysis: 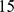 books in Portuguese, 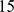 books in English, and 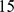 versions of the New Testament in different languages, see Supplementary Information S1 for details. The list of books in English and Portuguese is provided respectively in Table S1 and Table S2. We consider also the average 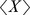 and the standard deviation 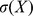 of 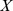 computed over different books (e.g., each of the three sets of 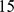 books) and the correlation 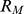 between 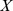 and the vocabulary size 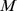 of the book. Table 1 shows the values of 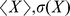 and 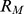 of all measurements in each of the three sets of books. In order to obtain further insights on the dependence of these measurements on language (syntax) and text (semantics), next we perform additional statistical analysis to identify measurements that are more suitable to target specific problems.

### Distinguishing Books from Shuffled Sequences

Our first aim is to identify measurements capable of distinguishing between natural and shuffled texts, which will be referred to as informative measurements. For instance, for 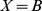 in Figure 1 all values are much smaller than 1 in all three sets of texts, indicating that this measurement takes smaller values in natural texts than in shuffled texts. In order to quantify the distance of a set of values 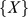 to 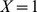 we define the quantity 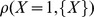 as the proportion of elements in the set 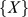 for which 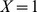 lies within the interval 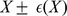, where 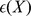 arises from fluctuations due to the randomness of the shuffling process (as defined in Eq. (8) below). This leads to condition 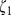:


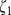
: 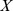 is said to be informative if 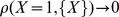 for 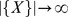,

where 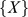 is a set of values 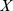 obtained over different texts in different languages or texts, and 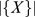 is the number of elements in this set.

We now discuss the results obtained applying 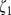 (with 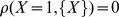) for all three sets of texts in our database for each of the measurements employed in this paper. Measurements which satisfied 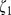 are indicated by a 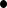 in Table 1. Several of the network measurements: the shortest path 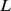 (i.e., the average shortest distance between two nodes), the diameter 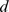 (i.e, the maximum shortest path), the clustering coefficient 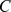 (i.e. the connectivity rate between neighbors of a network node), the average degree 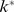 of the most frequent words and three small sub-graphs or network patterns (motifs 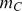, 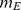 and 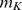) do not fully satisfy 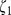. Consequently they cannot be used to distinguishing a manuscript from its shuffled version. This finding is rather surprising because some of the latter measurements were proven useful to grasp subtleties in text, e.g. for author recognition [4]. In the latter application, however, the networks representing text did not contain stopwords and the texts were lemmatized so that verbs and nouns were transformed into their infinitive and singular forms, respectively. When we performed the informativeness analysis over the most frequent words, we found that 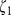 is satisfied for the clustering coefficient and for the shortest paths (note that 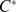 and 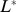 are informative while 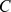 and 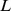 are not). This means that the informativeness of these quantities is concentrated in the most frequent words. On the other hand, for the degree, an opposite effect occurs, i.e., 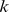 is informative and 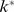 is not. The informativeness of intermittency (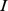 and 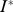) may be due to its definition as the *coefficient of variation* of the recurrence interval of words, which follows a Poisson distribution in shuffled texts. The mean and the variance of a Poisson distribution take the same values [24], then 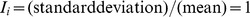 (see Materials and Methods). Since in natural texts many words tend to appear clustered in regions 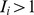 and 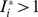. The selectivity 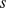, which quantifies the diversity of words appearing immediately before or after a given word, is also strongly affected by the shuffling process. Words in shuffled texts tend to be less selective, which yields an increase in 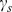 [25] (i.e., very selective words occur very sporadically) and a decrease in 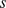 and 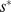. The selectivity is related to the effect of word consistency (see Ref. [26]) which was verified to be common in English, especially for very frequent words. The number of bigrams 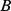 is also informative, which means that in natural languages it is unlikely that the same word is repeated (when compared with random texts). As for the informative motifs, 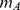, 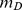, 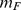, 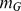, 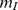, 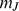, 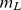 and 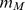 rarely occur in natural language texts (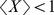) while motif 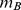 was the only measurement taking values above and below 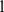. The emergence of this motif therefore appears to depend on the syntax, being very rare for Xhosa, Vietnamese, Swahili, Korean, Hebrew and Arabic.

### Dependence on Style and Language

We are now interested in investigating which text-measurements are more dependent on the language than on the style of the book, and vice-versa. Measurements depending predominantly on the syntax are expected to have larger variability across languages than across texts. On the other hand, measurements depending mainly on the story (semantics) being told are expected to have larger variability across texts in the same language. Note that this approach could be extended to account for different text genres, for distinct characteristics could be expected from novels, lyrics, encyclopedia, scientific texts, etc., i.e. 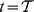. The variability of the measurements was computed with the coefficient of variation 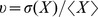, where 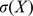 and 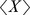 represent respectively the standard deviation and the average computed for the books in the set 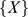. Thus, we may assume that 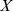 is more dependent on the language than on the style/semantics if condition 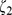 is satisfied:


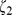
: 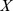 is more dependent on the language (or syntax) than it is on the style (or semantics) if 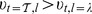.

Measurements failing to comply with condition 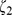 have 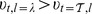 and therefore are more dependent on the style/semantics than on the language/syntax. In order to quantify whether 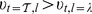 or 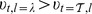 is statistically significant, we took the confidence interval of 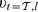 and 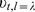. Let 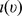 be the confidence interval for 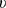 computed using the noncentral t-distribution [27], then 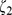 is valid if there is little intersection of the confidence intervals. In other words:


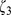
: The inequality 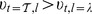 (or 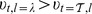) is valid only if 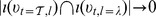 for 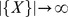.

In practice, the confidence intervals were assumed to have little intersection if 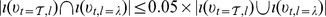. We took a significance level 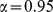 in the construction of the confidence intervals.

The results for the measurements satisfying conditions 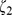 and 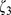 are shown in Table 1. Measurements satisfying conditions 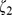 and 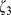 serve to examine the dependency on the syntax or on the style/semantics. The vocabulary size 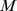, and the network measurements 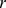 (assortativity or degree correlations between connected nodes), 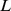 (shortest path length), 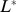, 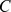 (clustering coefficient), 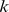 (degree) and 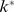 are more dependent on syntax than on semantics. The measurements derived from the selectivity (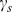, 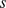 and 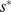) are also strongly dependent on the language. With regard to the motifs, five of them satisfy 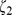 and 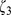: 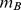, 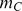, 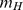, 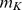 and 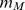. Remarkably, 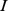 and 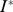 are the only measurements with low values of 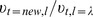. Reciprocally, the only measurement which statistically significantly violated 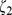 (i.e., satisfied 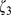) was 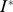. This confirms that the average intermittency of the most frequent words is more dependent on the style than on the language.

### On the Representativeness of Measurements

The practical implementation of our general framework was done quantifying the variation across languages using a single book (the New Testament). This was done because of the lack of available books in a large number of languages. In order for this approach to work it is essential to determine whether fluctuations across different languages are representative of the fluctuations observed in different books. We now determine the measurements 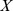 whose *actual values* of a single book on a specific language 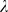 (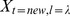) are compatible to other books in the same language (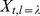). To this end we define the compatibility 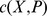 of 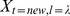 to 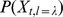. The distribution 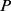 was taken with the Parzen-windowing interpolation [28] using a Gaussian function as kernel. More precisely, 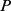 was constructed adding Gaussian distributions centered around each 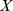 observed over different texts in a fixed language 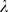. Mathematically, the compatibility 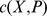 is computed as
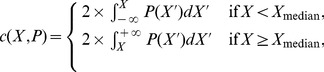
(1)where 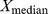 is the median of 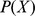. For practical purposes, we consider that 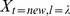 is compatible with other books written in the same language 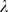 if 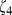 is fulfilled:


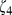
: 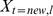 is a representative measurement of the language 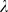 if 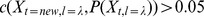.

Note that analogously to the methodology devised in Refs. [29], [30], 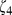 considers that a data element is an outlier if it is isolated from the other ones, which is revealed by a low probability of observing an element as extreme as the one considered outlier. The representativeness of the measurements computed for the New Testament was checked using the distribution 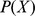 obtained from the set of books written in Portuguese and English. The standard deviation employed in the Parzen method was the least deviation between English and Portuguese, i.e. 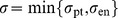. The measurements satisfying 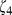 for both English and Portuguese datasets are displayed in the last column of Table 1. With regard to the network measurements, only 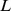, 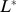, 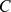 and 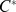 are representative, suggesting that they are weakly dependent on the variation of style (obviously assuming the New Testament as a reference). In addition, 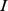, 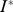, 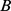, 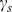, 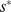 and 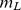 turned out to be representative measurements.

### Case Study: the Voynich Manuscript (VMS)

So far we have introduced a framework for identifying the dependency of different measurements on the language (see e.g. the second column of Table 1) and style/story of different books (see e.g. columns 3–4 of Table 1). We now investigate to which extent the measurements we identified as relevant can provide information upon analyzing single texts. The Voynich Manuscript (VMS), named after the book dealer Wilfrid Voynich who bought the book in the early 20th century, is a 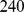 page folio that dates back to the 15th century. Its mysterious aspect has captivated people’s attention for centuries. Indeed, the VMS has been studied by professional cryptographers, being a challenge to scholars and decoders [31], [32], currently included among the six most important ciphers [31]. The various hypotheses about the VMS can be summarized into three categories: (i) A sequence of words without a meaningful message; (ii) a meaningful text written originally in an existing language which was coded (and possibly encrypted with a mono-alphabetic cipher) in the Voynich alphabet; and (iii) a meaningful text written in an unknown (possibly constructed) language. While it is impossible to investigate systematically all these hypotheses, here we perform a number of statistical analyses which aim at clarifying the feasibility of each of these scenarios. To address point (i) we analyze shuffled texts. To address point (ii) we consider 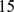 different languages, including the artificial language Esperanto that allows us to touch on point (iii) too. We do not consider the effect of poly-alphabetic encryption of the text because the whole statistical analysis would be influenced by the properties of encryption and thus the information about the “language of the VMS” would be lost.

The statistical properties of the VMS were obtained to try and answer the questions posed in Table 2, which required checking the measurements that would lead to statistically significant results. To check whether a given text is compatible with its shuffled version, 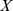 computed in texts written in natural languages should always be far from 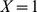, and therefore only informative measurements are able to answer question Q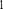. To test whether a text is consistent with some natural language (question Q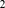), the texts employed as basis for comparison (i.e., the New Testament) should be representative of the language. Accordingly, condition 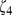 must be satisfied when selecting suitable measurements to answer Q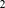. 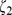 and 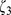 must be satisfied for measurements suitable to answer Q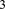 because the variability in style within a language should be small, if one wishes to determine the most similar language. Otherwise, an outlier text in terms of style could be taken as belonging to another language. An analogous reasoning applies to selecting measurements to identify the closest style. Finally, note that answers for Q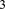 and Q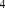 depend on a comparison with the New Testament in our dataset. Hence, suitable measurements must fulfill condition 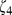 in order to ensure that the measurements computed for the New Testament are representative of the language.

**Table 2:** List of fundamental questions for identifying the nature of unknown manuscripts.

**Questions**		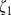	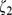	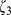	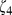
Q_1_	Is the text compatible with shuffled version?	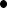			
Q_2_	Is the text compatible with a natural language?				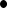
Q_3_	Which language is closer to the manuscript?		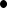	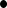	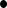
Q_4_	Which style is closer to the manuscript?			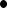	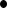

Conditions to be fulfilled by the measurements for answering each of the questions posed. Condition 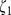 is useful for selecting informative metrics, since this condition ensures that shuffled texts can be distinguished from texts written in natural language. The metrics satisfying condition 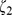 are useful to discriminate languages because the fulfillment of this condition ensures low variation attributed to semantic factors, and therefore discrimination depends on syntactic factors. Condition 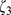 is useful to find the closest language/style because it is related to significance tests performed in 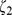. Finally, condition 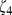 is useful to ensure that the metrics computed in the New Testament are representative of the language.

#### Is the VMS distinguishable from its shuffled text?

Before checking the compatibility of the VMS with shuffled texts, we verified if Q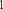 can be accurately answered in a set of books written in Portuguese and English, henceforth referred to as test dataset (see Table S3). A given test text was considered as not shuffled if the interval 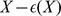 to 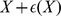 does not include 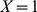. To quantify the distance of a text from its shuffled version, we defined the distance 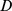:
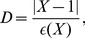
(2)which quantifies how many 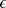
’s the value 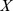 is far from 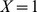. As one should expect, the values of 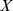 computed in the test dataset for 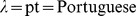 and 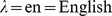 (see Table S4) indicate that no texts are compatible with their shuffled version because 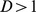, which means that the interval from 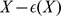 to 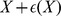 does not include 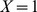. Since the methodology appropriately classified the texts in the test dataset as incompatible with their shuffled versions, we are now in a position to apply it to the VMS.

The values of 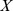 for the VMS, denoted as 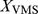, in Table 3 indicate that the VMS is not compatible with shuffled texts, because the interval from 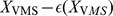 to 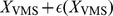 does not include 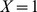. All but one measurement (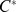) include 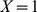 in the interval 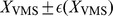, suggesting that the word order in the VMS is not established by chance. The property of the VMS that is most distinguishable from shuffled texts was determined quantitatively using the distance 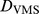 from Eq. (2). Table 3 shows the largest distances for intermittency (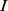 and 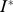) and network measurements (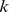 and 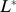). Because intermittency is strongly affected by stylistic/semantic aspects and network measurements are mainly influenced by syntactic factors, we take these results to mean that the VMS is not compatible with shuffled, meaningless texts.

**Table 3:** Analysis of compatibility of the VMS with shuffled texts.

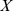	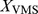 -	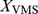	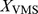 +	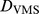
	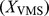		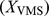	
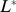	1.069	1.071	1.072	47
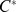	0.981	0.999	1.017	0
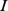	1.423	1.433	1.443	44
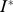	1.875	1.890	1.904	61
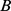	2.333	2.637	2.940	5
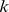	0.948	0.949	0.950	51
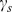	0.617	0.692	0.768	23
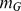	0.782	0.796	0.809	15
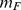	0.738	0.751	0.765	18
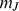	0.784	0.798	0.813	14
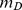	0.908	0.940	0.971	2
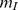	0.724	0.733	0.741	32
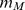	0.783	0.801	0.819	11
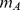	0.728	0.739	0.751	23
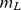	0.549	0.582	0.616	12

Values of 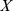 for the Voynich Manuscript considering only the informative measurements (i.e., the measurements satisfying 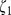). Apart from 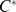 all measurements point to the VMS being different from shuffled texts.

#### Is the VMS compatible with a text in natural languages?

The compatibility with natural languages was checked by comparing the suitable measurements for the VMS with those for the New Testament written in 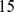 languages. Similarly to the analysis of compatibility with shuffled texts, we validated our strategy in the test dataset as follows. The compatibility with natural texts was computed using Eq. (1), where 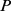 was computed adding Gaussian distributions centered around each 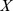 observed in the New Testament over different languages 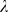. The standard deviation on each Gaussian representing a book in the test dataset should be proportional to the variation of 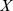 across different texts and therefore we used the least 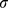 between English and Portuguese. The values displayed in Table S5 reveal that all books are compatible with natural texts, as one should expect. Therefore we have good indications the proposed strategy is able to properly decide whether a text is compatible with natural languages. The distance from the VMS to the natural languages was estimated by obtaining the compatibility 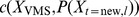 (see Eq. (1)).

**Figure 2:**
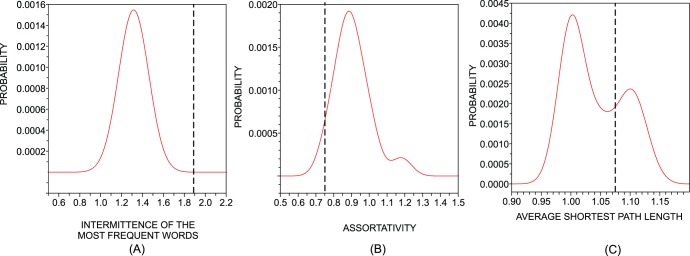
Distribution of measurements for the New Testament compared with the measurement obtained for VMS (dotted line). The measurements are (a) 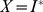 (intermittency of the most frequent words); (b) 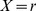 (assortativity) and (c) 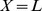 (average shortest path length). While in (a) VMS is not compatible with natural languages, in (b) and (c) the compatibility was verified since 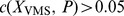.

The distribution 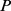 for three measurements is illustrated in Figure 2. The values of 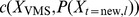 displayed in Table 4 confirm that VMS is compatible with natural languages for most of the measurements suitable to answer Q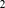. The exceptions were 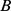 and 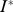. A large 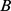 is a particular feature of VMS because the number of duplicated bigrams is much greater than the expected by chance, unlike natural languages. 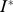 is higher for VMS than the typically observed in natural languages (see Figure 2(a)), even though the absolute intermittence value of the most frequent words in VMS is not far from those for natural languages. Since the intermittency 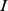 is related to large scale distribution of a (key) word in the text, we speculate that the reason for these observations may be the fact that the VMS is a compendium of different topics, which is also suggested by illustrations related to herbs, astronomy, cosmology, biology etc.

**Table 4:** Analysis of compatibility of the VMS with texts written in natural language.

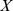	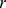	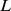	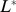	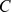	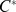	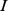	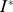	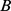	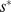	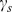
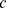	0.14	0.62	0.99	0.96	0.05	0.39	0.00	0.00	0.09	0.12

Compatibility of VMS with natural languages. Except for 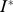 and 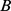, the measurements computed for VMS are consistent with those expected for texts written in natural languages.

#### Which language/style is closer to the VMS?

We address this question in full generality but we shall show that with the limited dataset employed, we cannot obtain a faithful prediction of the language of a manuscript. Given a text 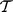, we identify the most similar language according to the following procedure. Each book is characterized by the measurements suitable to answer Q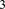 in Table 2. To avoid the different magnitudes of different measurements interfering with distinct weights in the calculation of similarity between books, we used the z-normalized values of the metrics. As such, the distance between the book 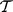 and a version of the New Testament written in the language 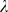 is given by:
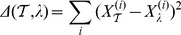
(3)where 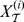 and 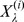 represent the i-th z-normalized measurement computed for 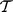 and 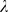, respectively. Let 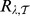 be the ranking obtained by language 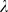 in the text 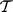 when 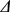 is sorted in ascending order. Given a set of texts 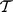 written in the same language, this procedure yields a list of 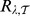 for each 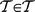. In this case, it is useful to combine the different 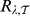 by considering the product of the normalized ranks
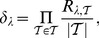
(4)where 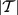 is the number of texts in the database 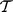. This choice is motivated by the fact that 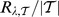 corresponds to the probability of achieving by chance a ranking as good as 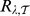 so that 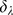 in Eq. (4) corresponds to the probability of obtaining such a ranking by chance in every single case. By ranking the languages according to 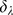 we obtain a ranking of best candidates for the language of the texts in 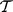.

In our control experiments with 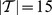 known texts we verified that the measurements suitable to answer Q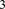 led to results for the books in Portuguese and English of our dataset which do not always coincide with the correct language. In the case of the Portuguese test dataset, Portuguese was the second best language (after Greek), while in the English dataset the most similar languages were Greek and Russian and English was only in place 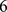. Even though the most similar language did not match the language of the books, the 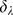 obtained were significantly better than chance (p-value = 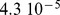 and 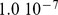, respectively in the English and Portuguese test sets).

The reason why the procedure above was unable to predict the accurate language of our test books in English and Portuguese is directly related to the use of only one example (a version of the New Testament) for each language, while in robust classification methods many examples are used for each class. Hence, finding the most similar language to VMS will require further efforts, with the analysis of as many as possible books representing each language, which will be a challenge since there are not many texts widely translated into many languages.

#### Keywords of the VMS

One key problem in information sciences is the detection of important words as they offer clues about the text content. In the context of decryption, the identification of keywords may be helpful for guiding the deciphering process, because cryptographers could focus their attention on the most relevant words. Traditional techniques are based on the analysis of frequency, such as the widely used term frequency-inverse document frequency [18] (tf-idf). Basically, it assigns a high relevance to a word if it is frequent in the document under analysis but not in other documents of the collection. The main drawback associated with this approach is the requirement of a set of representative documents in the same language. Obviously, this restriction makes it impossible to apply tf-idf to the VMS, since there is only one document written in this “language”. Another possibility would be to use entropy-based methods [5], [20] to detect keywords. However, the application of all these methods to cases such as the VMS will be limited because they typically require the manuscript to be arranged in partitions, such as chapters and sections, which are not easily identified in the VMS.

**Figure 3:**
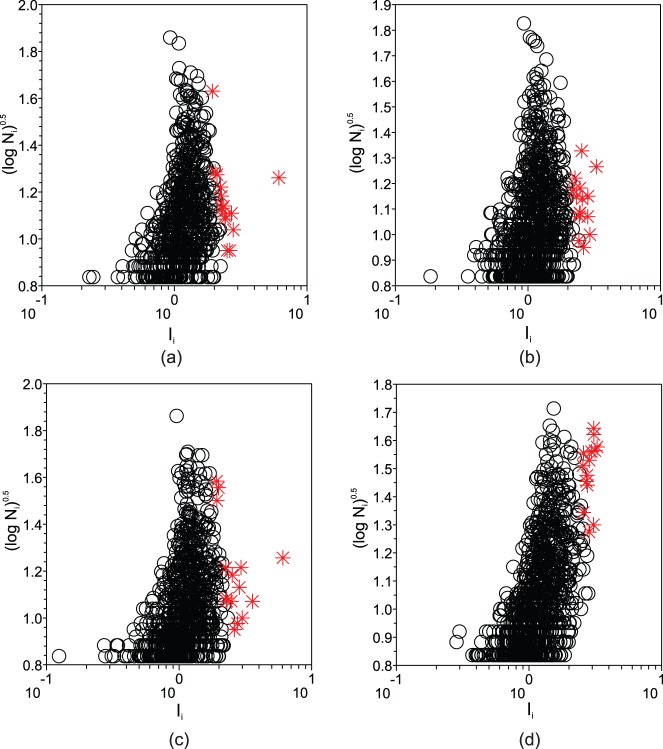
Keywords for the New Testament and for the Voynich manuscript. For the New Testament, the languages analyzed were (a) the Portuguese, (b) the English, and (c) the German. The list of keywords for the Voynich manuscript is shown in (d). 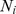 corresponds to the number of occurrences of the word 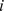 in the text and 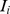 is the measure of intermittency defined in Eq. (6). The keywords are obtained from Eq. (5) and are marked by 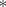, other words are indicated by circles. Note that keywords are characterized by high 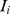 and high 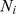. In all three languages the top keyword (corresponding to “begat” in English) can be explained by its concentration (large intermittency I) in the description of the genealogy of Jesus in two passages of the New Testament.

To overcome this problem, we use the fact that keywords show high intermittency inside a single text [5]–[7], [21]–[23]. Therefore, this feature can play the role traditionally played by the inverse document frequency (idf). In agreement with the spirit of the tf-idf analysis, we define the relevance 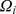 of word 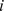 as
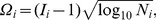
(5)where the intermittency 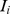 is defined in Eq. (6) and 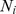 is the absolute number of occurrences of word 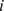. Alternative combinations of these two factors can be used depending on the specific application (e.g., for books with different sizes a term proportional to the normalized frequency could be used instead of 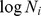). Note that with the factor 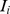, words with 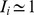 receive low values of 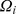 even if they are very frequent (large 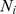). For the case of small texts and small frequency, corrections on our definition of intermittency should be used, see Ref. [7] which also contains alternative methods for the computation of keywords from intermittency. In order to validate 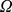 we applied Eq. (5) to the New Testament in Portuguese, English and German. Figure 3 illustrates the disposition of keywords with regard to the frequency and intermittency terms. An inspection of Table 5 for Portuguese, English and German indicates that representative words have been captured, such as the characters “Pilates”, “Herod”, “Isabel” and “Maria” and important concepts of the biblical background such as “nasceu” (was born), “cus”/” himmelreich” (heavens), “heuchler” (hypocrite), “demons” and “sabbath”. Interestingly, the keywords found for the three languages are not the same, in spite of the same contents in the book analyzed. This suggests that keywords may depend strongly on the translator. In fact, replacements of words with synonymous ones could easily turn a keyword into an “ordinary” word. Finally, in the right column of Table 5 we present the list of words obtained for the VMS through the same procedure, which are natural candidates as keywords.

**Table 5:** Keywords found for the New Testament and for the Voyninch manuscript.

Portuguese	English	German	Voynich
nasceu	begat	zeugete	cthy
Pilatos	Pilates	zentner	qokeedy
céus	talents	himmelreich	shedy
bem-aventurados	loaves	pilatus	qokain
Isabel	Herod	schwert	chor
anjo	tares	Maria	lkaiin
menino	vineyard	Elisabeth	qol
vinha	shall	Etliches	lchedy
sumo	boat	unkraut	sho
sepulcro	demons	euch	qokaiin
joio	five	schiff	olkeedy
Maria	pay	ihn	qokal
portanto	sabbath	weden	qotain
Herodes	hear	heuchler	dchor
talentos	whosoever	tempel	otedy

Keywords of the New Testament (English, Portuguese and German) and the VMS using Eq. (5).

### Conclusion

In this paper we have developed the first steps towards a statistical framework to determine whether an unknown piece of text, recognized as such by the presence of a sequence of symbols organized in “words”, is a meaningful text and which language or style is closer to it. The framework encompassed statistical analysis of individual words and then books using three types of measurements, namely metrics obtained from first-order statistics, metrics from networks representing text and the intermittency properties of words in a text. We identify a set of measurements capable of distinguishing between real texts and their shuffled versions, which were referred to as informative measurements. With further comparative studies involving the same text (New Testament) in 15 languages and distinct books in English and Portuguese, we could also find metrics that depend on the language (syntax) to a larger extent than on the story being told (semantics). Therefore, these measurements might be employed in language-dependent applications. Significantly, the analysis was based entirely on statistical properties of words, and did not require any knowledge about the meaning of the words or even the alphabet in which texts were encoded.

The use of the framework was exemplified with the analysis of the Voynich Manuscript, with the final conclusion that it differs from a random sequence of words, being compatible with natural languages. Even though our approach is not aimed at deciphering Voynich, it was capable of providing keywords that could be helpful for decipherers in the future.

## Materials and Methods

### Description of the Measurements

**Figure 4:**
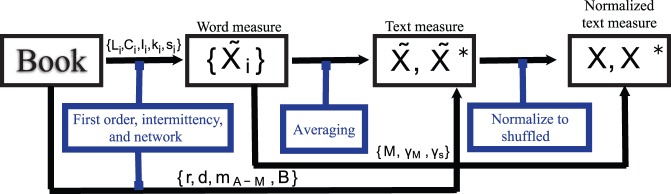
Illustration of the procedures performed to obtain a measurement 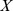 of each book.

The analysis involves a set of steps going beyond the basic calculation of measurements, as illustrated in the workflow in Figure 4. Some measurements are averaged in order to obtain a measurement on the text level from the measurement on the word level. In addition, a comparison with values obtained after randomly shuffling the text is performed to assess to which extent structure is reflected in the measurements.

#### First-order statistics

The simplest measurements obtained are the vocabulary size 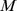, which is the number of distinct words in the text, and the absolute number of times a word 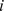 appears in a document), denoted by 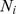. The heterogeneity of the contexts surrounding words was quantified with the so-called selectivity measurement [25]. If a word is strongly selective then it always co-occurs with the same adjacent words. Mathematically, the selectivity of a word 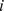 is 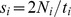, where 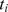 is the number of distinct words that appear immediately beside (i.e., before or after) 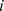 in the text.

A language-dependent feature is the number of different words (types) that at least once had two word tokens immediately beside each other in the text. In some languages this repetition is rather unusual, but in others it may occur with a reasonable frequency (see Results) and Figure 1). In this paper, the number of repeated bigrams is denoted by 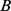.

#### Network characterization

Complex networks have been used to characterize texts [3], [4], [8], [9], [19], where the nodes represent words and links are established based on word co-occurrence, i.e. links between two nodes are established if the corresponding words appear at least once adjacent in the text. In other words, if word 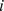 appears before word 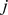 in a given document, then the arc 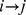 is established in the network. In most applications of co-occurrence networks, the stopwords (i.e., highly frequent words usually conveying little semantic information) are removed and the remaining words are transformed to their canonical form. Thus conjugated verbs and plural nouns are converted to their infinitive and singular forms, respectively. Here, we decided not to do this because in unknown languages it is impossible to derive lemmatized word forms or identify stopwords. To characterize the structure and organization of the networks, the following topological metrics of complex networks were calculated (more details are given in the SI).

- We quantify *degree correlations* (or assortativity), i.e. the tendency of nodes of certain degree to be connected to nodes with similar degree (the degree of a node is the number of links it has to other nodes), with the Pearson correlation coefficient, 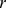, thus distinguishing assortative (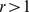) from disassortative (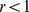) networks.
- The so-called clustering coefficient, 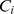, is given by the fraction of closed triangles of a node, i.e. the number of actual connections between neighbours of a node divided by the possible number of connections between them. The global *clustering coefficient* 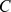 is the average over the local coefficients of all nodes.
- The *average shortest path length*, 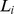, is the shortest path between two nodes 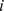 and 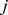 averaged over all possible 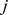
’s. In text networks it measures the relevance of words according to their distance to the most frequent words [4].
- The *diameter* 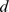 corresponds to the maximum shortest path, i.e. the maximum distance on the network between any two nodes.
- We also characterized the topology of the networks through the analysis of motifs, i.e. analysis of connectivity patterns expressed in terms of small building blocks (or subgraphs) [33]. We define as 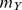 the number of motifs 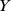 appearing in the network. The motifs employed in the current paper are displayed in Figure S1.

#### Intermittency

The fact that words are unevenly distributed along texts has been used to detect keywords in documents [5]–[7], [20]. Thinking the length of the text as a measure of time, such uneven distribution resembles a bursty or intermittent appearance (see, e.g., Ref. [21] and references therein). Words with different functions can be distinguished according to the degree of such intermittency, with keywords showing strong intermittent behavior (strong concentration in specific regions of the text). The uneven distribution of word-frequencies in time has recently been used also to identify external events through the analysis of large databases available in the Internet (see, e.g., Refs. [2], [34], [35] for recent examples).

The intermittency was calculated using the concept of recurrence times, which have been used to quantify the burstiness of time series [21], [23]. In the case of documents, the time series of a word is taken by counting the number of words (representing time) between successive appearances of the considered word. For example, the recurrence times for the word ‘the’ in the previous sentence are 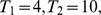 and 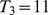. If 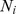 is the frequency of the word its time series will be composed by the following elements –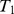, 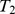, 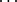 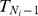}. Because the times until the first occurrence 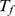 and after the last occurrence 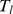 are not considered, the element 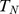 is arbitrarily defined as 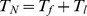. Note that with the inclusion of 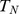 in the time series, the average value over all 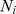 values is 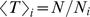. Then, to compute the heterogeneity of the distribution of a word 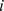 in the text, we obtained the intermittency 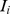 as
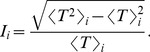
(6)Words distributed by chance have 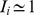 (for 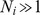), while bursty words have 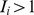. Words with 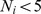 were neglected since they lack statistics.

Besides intermittency (or burstiness), long-range correlation is also used to characterize temporal properties of texts and complex systems in general (see, e.g., Refs. [22], [36] and references therein). We use intermittency because our analysis focuses on words while long-range correlation analysis typically use letters [32] (but see Ref. [22] for the relation between the different scales).

### From Word to Text Measurements

Many of the measurements defined in the previous section are attributes of the *word* 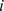. For our aims here it is essential to compare different *texts*. The easiest and most straightforward choice is to assign to a piece of text the average value of each measurement 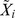, computed over all 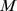 words in the text 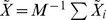. This was done for 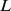, 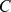, 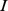, 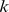 and 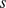. One potential limitation of this approach is that the same weight is attributed to each word, regardless of their frequency in the text. To overcome this, we also calculated another metric, 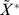 obtained as the average of the 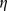 most frequent words, i.e. 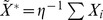, where the sum runs over the 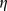 most frequent words. Here, we chose 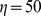. Finally, because 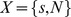 are known to have a distribution with long tails [18], [35], we also computed the scaling exponent 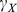 of the power-law 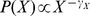, for which the maximum-likelihood methodology described in [37] was used.

### Comparison to Shuffled Texts

Since we are interested in measurements capable of distinguishing a meaningful text from its shuffled version, each of the measurements 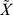 and 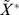 was normalized by the average obtained over 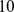 texts produced using a word shuffling process, i.e. randomizing preserving the word frequencies. If 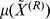 and 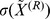 are respectively the average and the deviation over 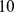 realizations of shuffled texts, the normalized measurement 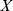 and the uncertainty 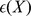 related to 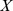 are:
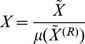
(7)

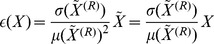
(8)


Normalization by the shuffled text is useful because it permits comparing each measurement with a null model. Hence, a measurement provides significant information only if its normalized 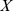 value is not 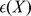 close to 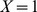. Moreover, the influence of the vocabulary size 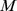 on the other measurements tends to be minimized.

## Supporting Information

Figure S1 **Illustration of 13 motifs comprising three nodes used to analyze the structure of text networks.**
(PDF)

Table S1 **List of Books in English.**
(TEX)

Table S2 **List of Books in Portuguese.**
(TEX)

Table S3 **Set of books in Portuguese and English employed to validate the methodology for checking the compatibility with shuffled and normal texts.**
(TEX)

Table S4
**Distance between original and shuffled texts.** If 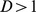 then the text is considered to be significantly different from its shuffled version.(TEX)

Table S5
**Values of compatibility with natural language manuscripts.** Texts are considered incompatible with natural languages whenever 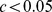.(TEX)

Supporting Information S1 (TEX)

## References

[1] Golder SA, Macy MW (2011) Diurnal and seasonal mood vary with work, sleep, and daylength across diverse cultures. Science 333: 1878–1881. 21960633 10.1126/science.1202775

[2] Michel JB, Shen YK, Aiden AP, Veres A, Gray MK, et al (2011) Quantitative analysis of culture using millions of digitized books. Science 331: 176–182. 21163965 10.1126/science.1199644 PMC3279742

[3] Amancio DR, Oliveira Jr ON, Costa LF (2012) Identification of literary movements using complex networks to represent texts. New J Phys 14: 043029.

[4] Amancio DR, Altmann EG, Oliveira Jr ON, Costa LF (2011) Comparing intermittency and network measurements of words and their dependence on authorship. New J Phys 13: 123024.

[5] Herrera JP, Pury PA (2008) Statistical keyword detection in literary corpora. EPJ B 63: 824–827.

[6] Ortuno M, Carpena P, Bernaola-Galvn P, Muoz E, Somoza AM (2002) Keyword detection in natural languages and dna. Europhys Lett 57: 759.

[7] Carretero-Campos C, Bernaola-Galvn P, Coronado A, Carpena P (2013) Improving statistical keyword detection in short texts: Entropic and clustering approaches. Physica A 392: 1481–1492.

[8] Ferrer i Cancho R, Solé RV, Köhler R (2004) Patterns in syntactic dependency networks. Phys Rev E Stat Nonlin Soft Matter Phys 69: 051915. 15244855 10.1103/PhysRevE.69.051915

[9] Ferrer i Cancho R, Solé RV (2001) The small world of human language. Proc R Soc B 268: 2261–2265. 10.1098/rspb.2001.1800 PMC1088874 11674874

[10] Petersen AM, Tenenbaum JN, Havlin S, Stanley HE (2012) Statistical laws governing uctuations in word use from word birth to word death. Sci Rep 2. 10.1038/srep00313 PMC3304511 22423321

[11] Petersen AM, Tenenbaum JN, Havlin S, Stanley HE, Perc M (2012) Languages cool as they expand: Allometric scaling and the decreasing need for new words. Sci Rep 2. 10.1038/srep00943 PMC3517984 23230508

[12] Singhal A (2001) Modern information retrieval: A brief overview. Bulletin of the IEEE Computer Society Technical Committee on Data Engineering 24: 35–43.

[13] Croft B, Metzler D, Strohman T (2009) Search Engines: Information Retrieval in Practice. Addison Wesley, 1 edition.

[14] Koehn P (2010) Statistical Machine Translation. Cambridge University Press, 1 edition.

[15] Amancio DR, Antiqueira L, Pardo TAS, Costa LF, Oliveira Jr ON, et al (2008) Complex network analysis of manual and machine translations. Int J Mod Phys C 19: 583–598.

[16] Yatsko V, Starikov MS, Butakov AV (2010) Automatic genre recognition and adaptive text summarization. In: Automatic Documentation and Mathematical Linguistics. 111–120.

[17] Nirenburg S (1989) Knowledge-based machine translation. Machine Translation 4: 5–24.

[18] Manning CD, Schutze H (1999) Foundations of Statistical Natural Language Processing. Cambridge, MA: MIT.

[19] Masucci AP, Rodgers GJ (2006) Network properties of written human language. Phys Rev E Stat Nonlin Soft Matter Phys 74: 026102. 17025498 10.1103/PhysRevE.74.026102

[20] Montemurro MA, Zanette DH (2001) Entropic analysis of the role of words in literary texts. Adv Complex Syst 5.

[21] Altmann EG, Pierrehumbert JB, Motter AE (2009) Beyond word frequency: bursts, lulls, and scaling in the temporal distributions of words. PloS ONE 4: e7678. 19907645 10.1371/journal.pone.0007678 PMC2770836

[22] Altmann EG, Cristadoro G, Esposti MD (2012) On the origin of long-range correlations in texts. Proc Natl Acad Sci USA 109: 11582–11587. 22753514 10.1073/pnas.1117723109 PMC3406867

[23] Serrano MA, Flammini A, Menczer F (2009) Modeling statistical properties of written text. PLoS ONE 4: e5372. 19401762 10.1371/journal.pone.0005372 PMC2670513

[24] Ross SM (2009) Introduction to probability models. Academic Press, 10 edition.

[25] Masucci AP, Rodgers GJ (2009) Differences between normal and shu_ed texts: structural properties of weighted networks. Adv Complex Syst 12: 113–129.

[26] Amancio DR, Oliveira Jr ON, Costa LF (2012) Using complex networks to quantify consistency in the use of words. J Stat Mech Theor Exp 2012: P01004.

[27] McKay AT (1932) Distribution of the coe_cient of variation and the extended t distribution. Jour Roy Stat Soc 95: 695–698.

[28] Parzen E (1962) On estimation of a probability density function and mode. Ann Math Stat 33: 1065–1076.

[29] Echtermeyer C, Costa LF, Rodrigues FA, Kaiser M (2011) Automatic network _ngerprinting through single-node motifs. PLoS ONE 6: e15765. 21297963 10.1371/journal.pone.0015765 PMC3031529

[30] Costa LF, Rodrigues FA, Hilgetag CC, Kaiser M (2009) Beyond the average: detecting global singular nodes from local features in complex networks. Europhys Lett 87: 18008.

[31] Belfield R (2007) The Six Unsolved Ciphers. Ulysses Press.

[32] Schinner A (2007) The voynich manuscript: Evidence of the hoax hypothesis. Cryptologia 31: 95–107.

[33] Milo R, Shen-Orr S, Itzkovitz S, Kashtan N, Chklovskii D, et al (2002) Network motifs: simple building blocks of complex networks. Science 298: 824–827. 12399590 10.1126/science.298.5594.824

[34] Klimek P, Bayer W, Thurner S (2011) The blogosphere as an excitable social medium: Richter’s and omori’s law in media coverage. Physica A 390: 3870–3875.

[35] Sano Y, Yamada K, Watanabe H, Takayasu H, Takayasu M (2013) Empirical analysis of collective human behavior for extraordinary events in the blogosphere. Phys Rev E Stat Nonlin Soft Matter Phys 87: 012805. 23410386 10.1103/PhysRevE.87.012805

[36] Rybski D, Buldyrev SV, Havlin S, Liljeros F, Makse HA (2009) Scaling laws of human interaction activity. Proc Natl Acad Sci USA 106: 12640–12645. 19617555 10.1073/pnas.0902667106 PMC2722366

[37] Clauset A, Shalizi CR, Newman MEJ (2009) Power-law distributions in empirical data. SIAM Rev 51: 661–703.

