# Supplementary material for: Probing the Statistical Properties of Unknown Texts: Application to the Voynich Manuscript
Source: PLoS One. 2013 Jul 2;8(7):e67310. doi: 10.1371/journal.pone.0067310 (PMC3699599; doi:10.1371/journal.pone.0067310)
Supplement: Figure S1 — Illustration of 13 motifs comprising three nodes used to analyze the structure of text networks. (PDF) [file pone.0067310.s001.pdf]

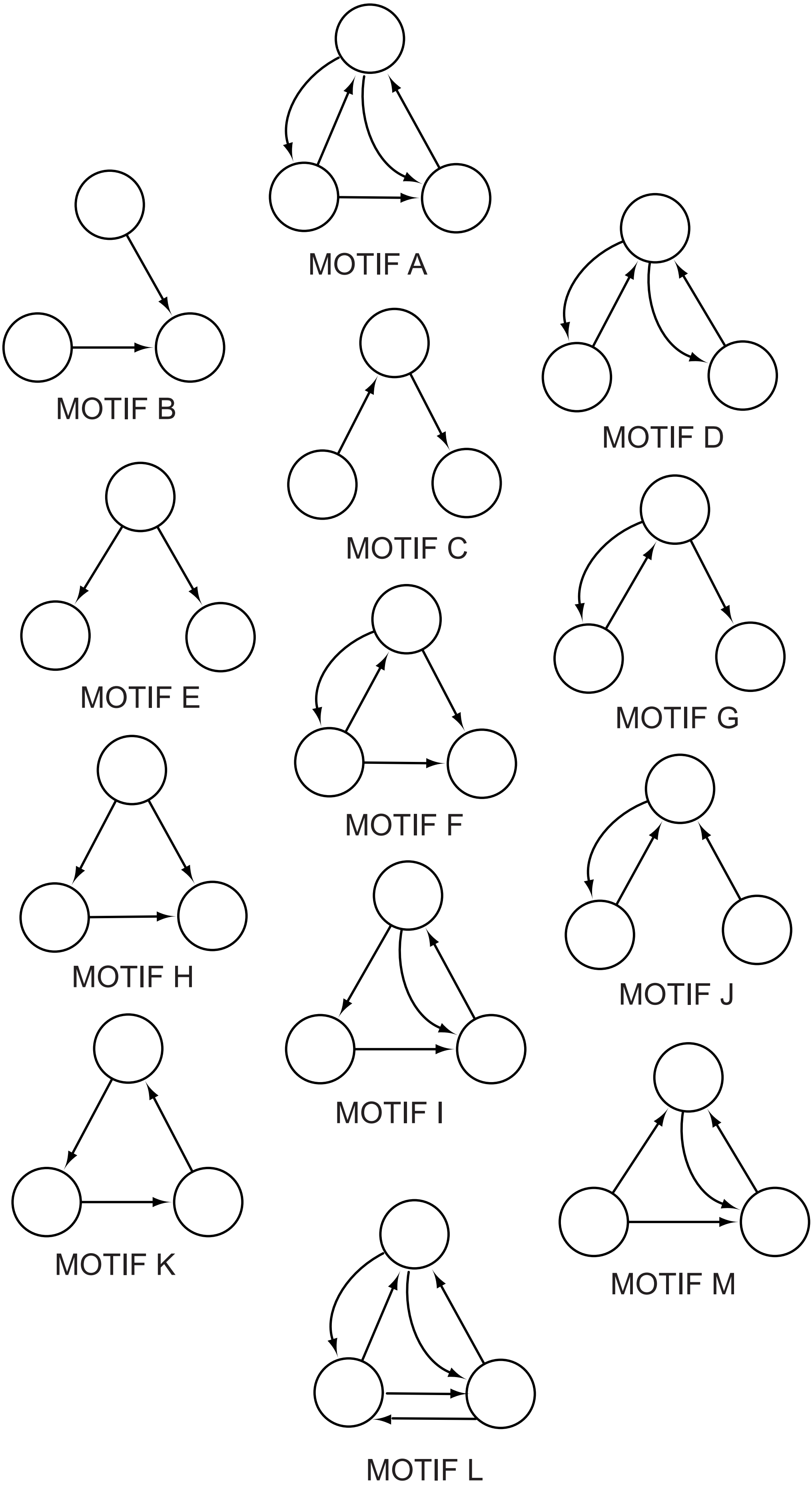
MOTIF A
MOTIF B
MOTIF D
MOTIF C
MOTIF E
MOTIF G
MOTIF F
MOTIF H
MOTIF J
MOTIF I
MOTIF K
MOTIF M
MOTIF L
